# Reducing geographic inequalities in access times for acute treatment of myocardial infarction in a large country: the example of Russia

**DOI:** 10.1093/ije/dyy146

**Published:** 2018-08-01

**Authors:** Sergey Timonin, Anna Kontsevaya, Martin McKee, David A Leon

**Affiliations:** 1 International Laboratory for Population and Health, National Research University Higher School of Economics, Moscow, Russia; 2 Research Laboratory of the Economic Analysis of the Epidemiology Surveys and Preventive Programs, National Research Center for Preventive Medicine of the Ministry of Healthcare of the Russian Federation, Moscow, Russia; 3 Department of Health Services Research and Policy, London School of Hygiene and Tropical Medicine, London, UK; 4 Department of Non-communicable Disease Epidemiology, London School of Hygiene and Tropical Medicine, London, UK; 5 Department of Community Medicine, The Arctic University of Norway, Tromsø, Norway

**Keywords:** percutaneous coronary intervention (PCI), myocardial infarction (MI), travel access, driving times, Russia

## Abstract

**Background:**

Russia has the largest area of any country in the world and has one of the highest cardiovascular mortality rates. Over the past decade, the number of facilities able to perform percutaneous coronary interventions (PCIs) has increased substantially. We quantify the extent to which the constraints of geography make equitable access to this effective technology difficult to achieve.

**Methods:**

Hospitals performing PCIs in 2010 and 2015 were identified and combined with data on the population of districts throughout the country. A network analysis tool was used to calculate road-travel times to the nearest PCI facility for those aged 40+ years.

**Results:**

The number of PCI facilities increased from 144 to 260 between 2010 and 2015. Overall, the median travel time to the closest PCI facility was 48 minutes in 2015, down from 73 minutes in 2010. Two-thirds of the urban population were within 60 minutes’ travel time to a PCI facility in 2015, but only one-fifth of the rural population. Creating 67 new PCI facilities in currently underserved urban districts would increase the population share within 60 minutes’ travel to 62% of the population, benefiting an additional 5.7 million people currently lacking adequate access.

**Conclusions:**

There have been considerable but uneven improvements in timely access to PCI facilities in Russia between 2010 and 2015. Russia has not achieved the level of access seen in other large countries with dispersed populations, such as Australian and Canada. However, creating a relatively small number of further PCI facilities could improve access substantially, thereby reducing inequality.

**Key Messages**

- Whereas rapid, invasive management has transformed the management of acute myocardial infarction (AMI), large countries with unevenly distributed populations, such as Russia, face challenges in delivering it equitably.
- The number of hospitals performing percutaneous coronary interventions (PCIs) in Russia almost doubled between 2010 and 2015 (from 144 up to 260), demonstrating substantial progress in providing advanced medical treatment for patients with AMI.
- In 2015, about half of all Russian adults aged 40+ years lived within 1 hour’s travel time to the closest PCI facility, with a pronounced geographical inequality particularly between urban and rural populations. However, the creation of 67 further PCI facilities in currently underserved urban districts would significantly improve overall access, enabling Russia to approach Australia and Canada in terms of the share of people living within 60 minutes’ travel access.
- For regions with small, sparsely populated settlements, it may be better to deploy advanced support in vehicles or non-PCI hospitals with subsequent transportation to PCI facilities by road or air if needed.

## Introduction

The management of many common medical emergencies (i.e. trauma, stroke, acute coronary syndrome) has been transformed in recent decades by technological, pharmacological and organizational advances. These share the need to get the patient to appropriately skilled and equipped staff and facilities within a short period of time.^1^^,^^2^ Primary percutaneous coronary intervention (PCI), the subject of this paper, is the preferred reperfusion strategy for patients who have suffered a myocardial infarction (MI).^3^ Yet, to be successful, treatment must be initiated rapidly.^4^^,^^5^ Current American and European guidelines recommend that relief of the blockage in the coronary artery should take place no more than 90–120 minutes after the onset of symptoms.^6^^,^^7^ However, there are many factors that might delay the patient’s journey from symptoms to intervention. Some delays may be attributable to the patient’s failing to recognize the potential seriousness of the symptoms and thus hesitating to call for help. Then there are delays related to travel time to the PCI facility, with distance being a major challenge in countries where the population is distributed unevenly over large areas, such as parts of the USA, Canada, Australia or Russia. Finally, delays can occur once the patient reaches the hospital or intervention centre.

National planning of an equitable reperfusion service must thus take into account the geographic dispersion of a country’s population. Studies in the USA,^8^ Australia^9^ and Canada^10^ have calculated the proportion of adults who could potentially be transferred to the closest PCI facility within a defined period of time if it was necessary. In the USA, nearly 80% of the adult population lived within 60 minutes of a PCI facility in 2000. Approximately 68% of persons aged 55+ years in Australia lived within 60 minutes’ travel time of acute and rehabilitative cardiac services in 2006. However, these individuals were concentrated in only 18% of administrative areas, reflecting the extremely uneven distribution of the Australian population. Research from Canada found that approximately 64% of people aged 40 years and older had timely access to PCI centres. However, in each country, there were huge regional variations in access to PCI facilities.

Recognizing Russia’s very high death rate from cardiovascular disease, which has persisted despite declines since the mid-2000s,^11^^,^^12^ the Russian government launched a major federal programme to improve health with a particular emphasis on cardiovascular disease.^13^ This included improving availability of interventional cardiology and, specifically, PCI. Its implementation was associated with large increases in numbers of procedures undertaken, with PCIs for acute coronary syndrome increasing from about 1500 to about 100 000 annually from 2001 to 2015, respectively.^14^ Further details of recent trends in the management of MI in Russia are provided in Box 1.

Box 1. Contemporary management of myocardial infarction in the Russian Federation

Thrombolysis can be administrated in almost all central district hospitals in Russia as well as by trained ambulance doctors and feldshers. According to official statistics provided by the Federal Ministry of Health, between 2010 and 2015, the percentage of patients with myocardial infarction having in-hospital thrombolysis within 12 hours of admission did not change significantly, from 26.4 to 25.8%, respectively, whereas the share of PCIs increased greatly, from 8.2% in 2010 to 41.6% in 2015. The share of patients who received thrombolysis in an ambulance also increased, from 2.8 to 6.6% over the same time period. Surprisingly, there is almost no difference between the share of urban and rural patients with myocardial infarction who received thrombolysis in an ambulance, at 6.7 and 6.2%, respectively. In 2015, thrombolysis was performed 13 632 times at the pre-hospital stage.

So far, much of this investment has been in existing clinics and hospitals in large population centres that had the capacity to implement PCI services. As further investments take place, it will be important to ascertain priorities for expansion to ensure more equitable geographical coverage, where possible, given the sparseness of the population in many parts of the country. To our knowledge, there has been no systematic attempt to assess the scale and nature of the geographical obstacles to ensuring equitable and timely access to PCI facilities in Russia.

In this study, we estimated the distances, expressed in road-driving times, to the closest hospital offering PCI across the territory of Russia, to ascertain the share of the population that might, in ideal circumstances, have rapid access to a centre if required. Furthermore, we explored the impact of expansion to cover currently underserved areas.

## Data and methods

### Data sources

Hospitals offering PCI in the Russian Federation were identified from the 2010 and 2015 editions of the bulletin published annually by the A.N. Bakoulev Scientific Centre for Cardiovascular Surgery.^14^ Using hospital names, we identified the addresses and geographical coordinates of each and generated a spatial dataset for mapping and modelling using a Geographic Information System (GIS).

**Figure 1.**
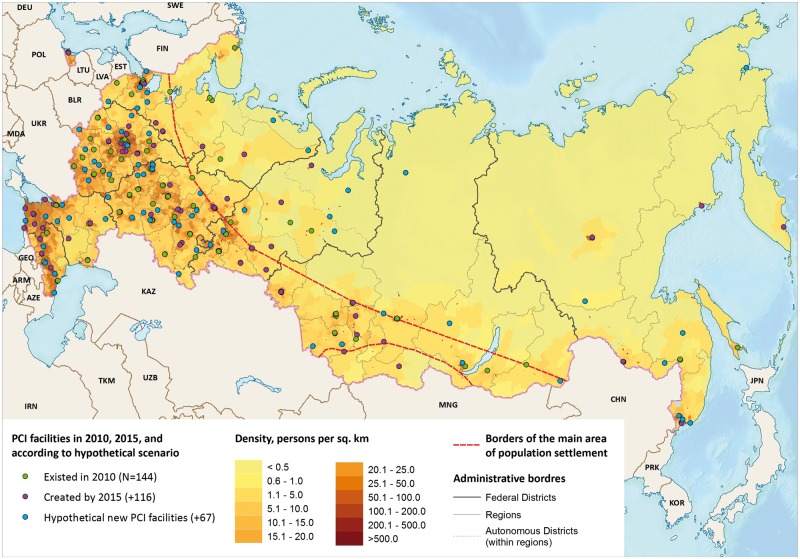
Location of PCI facilities in Russia in 2010 and 2015, and according to a scenario of creating 67 new hypothetical PCI facilities.

Population estimates were obtained from the 2010 Russian census provided by the Russian Federal State Statistics Service (Rosstat). We used age-specific data on the population of 2577 districts of Russia in the 83 regions of the Russian Federation. For simplicity, we use the word ‘region’ to describe all the upper-tier geographical entities, although they have a variety of names in Russian, including oblast and republic, relating to their degree of autonomy. Similarly, the term ‘district’ is used to include all second-tier entities, which again have a variety of names, depending mainly on whether they are urban or rural. The districts are the smallest geographical units at which census data are in the public domain. They include 236 intra-city districts in Moscow and St. Petersburg, with a mean population of 69 420; 516 urban districts, with a mean population of 131 560; and 1825 municipal districts, with a mean population of 32 100. Intra-city districts and urban districts have, as implied, mainly urban populations (100 and 97%, respectively), whereas municipal districts are mixed, with an average of 60% of the population in rural settings. These three types of districts also vary considerably in area. On average, urban districts are 10 times larger than intra-city districts and 10 times smaller than municipal districts. The uneven distribution of population across the country in terms of persons per square kilometre is shown in Figure 1. Almost 85% of the population lives in the 78% districts covering just 22% of the territory of Russia.

The geographical modelling used three spatial datasets. The first dataset contained polygons demarcating districts (*N* = 2577) covering the whole country. The second dataset contained points representing houses in cities with populations of greater than 1 million people. The third integrated dataset contained the network of roads in Russia.

The primary source of the first two datasets was OpenStreetMap (OSM), a crowd-based source of geographic data worldwide, including Russia. The district data were carefully checked for consistency and topology, and modified by authors where necessary. The dataset of houses in large cities was used as provided.

The road network graph was provided by the HERE company. HERE data have major advantages over other providers of data in Russia: the road mapping is topologically correct and covers the whole country, it represents the actual status of the road network and is presented in a ready-to-use format for modelling. The road network consists of numerous segments (edges) connected by nodes. For each segment, an average speed is estimated based on factors such as road class, results of GPS traffic measurements and speed assessment by field teams. The entire analysis was performed in ArcGIS 10.1 (ESRI).

### Modelling population travel times to PCI facilities

We estimated road-travel times from districts to PCI facilities by constructing routes from the physical centroids, i.e. geometric centres, of each district to the hospitals. We calculated median travel time by ranking districts served by a hospital in ascending order by travelling time and then calculating the cumulative population share contributed by each district. Median time was obtained when 50% of the cumulative population was reached. The same approach was used for 25th and 75th percentiles.

We modelled three scenarios. The first was for 2010 (number of PCI facilities = 144), the second for 2015 (*n* = 260) and, for the third (*n* = 327), we added an additional 67 hypothetical new PCI facilities to the 2015 scenario in currently underserved urban districts. This was to evaluate the impact on timely accessibility. We placed 63 of these hypothetical additional PCI facilities in cities with populations of 75 000 and over where the driving time to the closest existing PCI facility was more than 60 minutes. The other four PCI hypothetical facilities were placed in regional capitals that did not perform any PCIs.

We made several refinements to take account of settings where the geography might provide a misleading estimate of driving times. First, if the physical centroid of a district is 25 km or more from the nearest road segment, we used the location of the largest (by population size) settlement in the district to approximate its centre. Second, in urban districts with a population of more than 1 000 000 (except Moscow and St. Petersburg divided into intra-city districts), we performed additional calculations to estimate travelling time from each house in the city to the nearest PCI centre. The average value from these calculations is used as the final travelling time for each of these urban districts. However, a sensitivity analysis showed that these adjustments had only a minor impact on our main outcomes. Almost everyone in large cities was within 60 minutes’ travelling time anyway and almost no one in sparsely populated rural districts lived within 60–120 minutes’ travel distance.

In reality, in the majority of cases, people are transported to the nearest PCI facility in the region in which they live because of purely administrative preferences. This is despite the fact that there may be a closer one in an adjacent administrative region. We explored differences in population access times to a PCI centre according to whether all patients would travel to either the closest centre (in travel time) regardless of administrative borders or only went to the nearest centre in the region in which they lived. Our hypothesis is that individuals in some peripheral districts would have more rapid access to a facility in a neighbouring region rather than the one in which they live (see Supplementary Figure 1, available as Supplementary data at *IJE* online, for illustration). 

We report our results for the adult population of Russia aged 40+ years, as the risk of suffering an acute myocardial infarction (AMI) is very low at younger ages. We report driving times for each district, median driving times for larger administrative units (i.e. regions and federal districts) and Russia as a whole, and the proportion of the population aged 40+ years who had access to a PCI facility within 1 and 2 hours.

## Results

Between 2010 and 2015, the number of hospitals performing PCIs almost doubled from 144 to 260. This was accompanied by an increase in procedures performed in patients with AMI from 12 950 to 71 180 over the same period and by more regions providing advanced medical treatment.^14^Figure 1 shows the spatial location of the facilities in 2010, those added in the period to 2015 and the 67 hypothetical new facilities. Only one region in the European North, two in the Far East and one in the North Caucasus had no PCI facilities in 2015, whereas, in 2010, one-quarter of the Russian regions had no PCI facilities on their territory.

In 2015, the median travel time to the closest PCI facility by road for adults aged 40+ years was 48.2 minutes, down from 73.2 minutes in 2010 (Table 1). About half of all adults aged 40+ years lived within 1 hour’s and three-quarters were within 2 hours’ travel time. PCI was more easily accessible for those in urban districts, where 66% were within 60 minutes’ travel time, than rural districts, where the figure was only 20%. When using 2 hours’ travel time as a criterion, the difference between urban and rural residents is less pronounced (82 vs 56%).

**Table 1.** Median time to reach a PCI facility and share of population living within 60 or 120 minutes, or with no road access, for urban and rural settings in 2010 and 2015

Population	Adults aged 40+, mln	2010				2015			
		Median time (IQR), mins	60 min access, %	120 min access, %	No road access, %	Median time (IQR), mins	60 min access, %	120 min access, %	No road access, %
Total	67.5	73.2	45.1	66.0	0.6	48.2	53.9	75.3	0.3
		(13.5–155.9)				(9.3–118.6)			
Urban	49.7	30.8	56.2	74.3	0.6	15.4	66.0	82.3	0.3
		(10.2–122.6)				(7.3–88.9)			
Rural	17.7	137.0	14.3	42.9	0.5	109.3	20.3	56.2	0.3
		(84.7–209.7)				(68.8–173.0)			

PCI, percutaneous coronary intervention; IQR, interquartile range.

**Figure 2.**
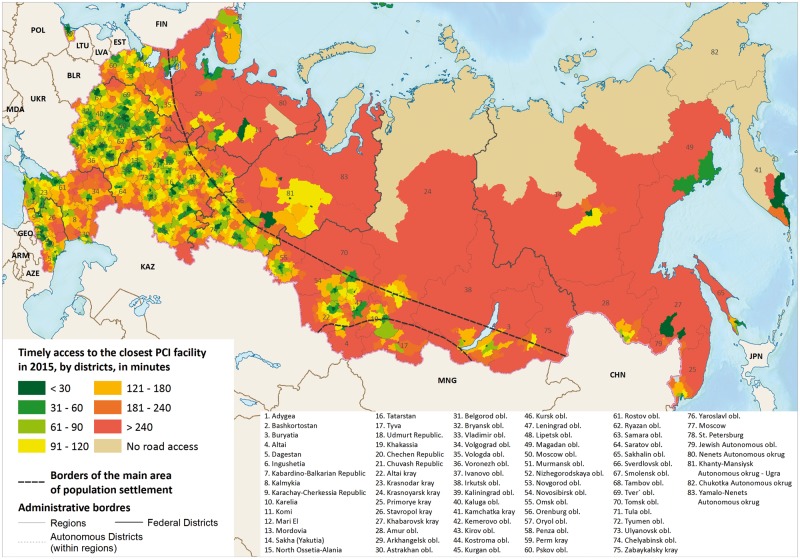
Driving times to the closest PCI facility in Russia in 2015.

Figure 2 shows the distribution of travel times to the nearest PCI facility. Whereas many districts in the centre of European Russia lie within 60–120 minutes’ travel time, this is not the case in very large parts of the country. In the Far East, many district centres are over 4 hours away from a PCI facility, whereas, in the arctic and near arctic, many have no road connection (approximately 200 000 people or 0.3% of those aged 40+ years in 40 local areas in 2015). Yet, whereas poor access is to be expected in rural, sparsely populated districts for PCI facilities, some populated cities and surrounding areas also lack adequate access to hospitals performing PCI.

In 2015, seven federal districts (groupings of regions) fell into three groups in terms of availability of PCI facilities: the central and north-western parts of European Russia, where access is better than the national average; the Volga region, which resembles the average for Russia as a whole; and other regions where access is worse (Supplementary Table 1, available as Supplementary data at *IJE* online). Whereas poor access to PCI facilities in Siberia and the Far East can be explained largely by difficulties created by geography, this does not explain the shortage of PCI facilities in the populated southern regions of European Russia. Progress between 2010 and 2015 in providing timely access to PCI facilities was observed in all federal districts, but the slowest improvements were in the Urals. Supplementary Figure 2, available as Supplementary data at *IJE* online, shows the proportion of adults aged 40+ years living within 60 minutes’ travel time to PCI facilities for the 83 regions of Russia.

The results shown so far have estimated travel times to the nearest PCI facility regardless of which region it was in. However, the financing and delivery of health care are largely devolved to the regions, so patients will normally be treated in the region in which they reside. For some people, the obligation to be treated in their home region might increase travel times compared with going to a facility that is closer but in another region. Consequently, we undertook a sensitivity analysis to compare differences in travel times to the nearest PCI facility in the same region or to the nearest facility wherever it is. This showed that, in 2015, 7 441 200 people or 11% of the population aged 40+ years lived in 400 bordering districts where it would be faster to go to neighbouring regions for treatment. For another 231 500 people who lived in 15 districts, PCI facilities are located only in neighbouring regions (Supplementary Figure 3, available as Supplementary data at *IJE* online). However, this option is associated with a relatively small increase of about 0.5% (337 690 people) in those who would live within 60 minutes’ travel time and 2.3% (1 456 130 people) within 120 minutes’ travel time.

Finally, we examined the effect of hypothetically creating 67 additional PCI facilities in currently underserved urban districts and in regions without any PCI facilities (Figure 1). For Russia as a whole, the creation of these new facilities would increase the population share within 60 minutes’ travel by 8.3% points (8.5% points for urban and 7.7% points for rural), to 62.1% of the population, benefiting 5.7 million adults aged 40+ years currently lacking adequate access (Table 2). Within Russia, the benefits would be greatest in the southern part of European Russia and the Far East (Supplementary Table 2 and Figure 4, available as Supplementary data at *IJE* online).

**Table 2.** Consequences for travel times (median travel time and share of population within 60 and 120 minutes) to PCI facilities of adding 67 new facilities, comparing estimated changes with actual times in 2015

Population	Adults aged 40+, mln	Hypothetical conditions (adding 67 new PCI facilities)			Changes in comparison with 2015			
		Median time (IQR), mins	60 min access, %	120 min access, %	Number of added facilities	Decrease in median time, mins	Increase in 60 min access, percent points	Increase in 120 min access, percent points
Total	67.5	34.7	62.1	83.6	67	14.1	8.3	8.2
		(9.0–90.4)						
Urban	49.7	13.5	74.5	89.5	67	1.9	8.5	7.2
		(7.0–61.9)						
Rural	17.7	88.4	28.0	67.2	0	20.9	7.7	11.0
		(56.3–139.5)						

PCI, percutaneous coronary intervention; IQR, interquartile range.

## Discussion

Our analyses have shown that the substantial growth in the number of PCI facilities in Russia between 2010 and 2015 has achieved an appreciable improvement in access to PCI, but substantial geographic inequalities persist. Even in 2015, we estimate that nearly half of the population lived more than 60 minutes from the nearest PCI facility, and a quarter more than 120 minutes distant.

Some degree of inequality in access times is perhaps inevitable given the size of Russia and the uneven dispersion of the population. However, today, the gaps are remain worryingly large. Those living in urban districts have appreciably better access than the 18 million rural residents, only one in five of whom had access within 60 minutes in 2015. These inequalities between urban and rural populations actually increased between 2010 and 2015. The best-served regions are those in the central part of European Russia, with the Far East having the poorest access of all, with less than 40% of residents having access in 60 minutes or less (Supplementary Table 1, available as Supplementary data at *IJE* online).

Whereas the economic case for creating new PCI facilities in highly dispersed areas of the Far East needs further investigation, expansion of facilities in the urban districts of the Russian South and Urals should be regarded as uncontentious (Supplementary Table 2, available as Supplementary data at *IJE* online). We estimate that, if another 67 facilities were added in populated but poorly served urban districts, there would be almost the same improvement in the share of the total population with access within 60 minutes (+8.3% points) as was achieved with the larger initial expansion of facilities (+8.8% points) between 2010 and 2015. These analyses provide *prima facie* evidence that Russia has not yet reached a point in increasing provision where potential benefits would show diminishing returns.

It is not currently possible to transport patients routinely to PCI facilities located in neighbouring regions, for administrative and financial restrictions. However, our analysis shows that, if all acute cases were taken to the nearest PCI facility regardless of whether it was in their region of residence or an adjacent one, there would be a measurable, although not substantial, overall impact.

The study has several limitations. We assessed travel times only by road and assuming good conditions that, at night and during winter months, are unlikely to be realized. Although it is beyond the scope of this analysis, the quality of the road network should also be considered. There are particular challenges in some parts of Russia because of seasonal melting of permafrost, coupled with other weather-related damage, but evidence from other countries has found that a better transport infrastructure can improve access to essential care.^15^ In some cases, helicopters or aircraft may be used, although, as a Norwegian review noted, these also face many challenges, especially weather conditions.^16^ Meanwhile, the Russian government initiated a new priority project in healthcare: ‘Development of sanitary aviation’.^17^ The aim is to increase the volume of emergency medical care to people living in hard-to-reach areas of the country. It envisages expenditure from the federal budget of 3.3 billion roubles (US$56 million) in 34 regions in 2017–20. However, this is unlikely to have a substantial impact on population access times for the country as a whole.

Second, our estimates make the simplistic assumption that the travel distances to the closest PCI facility are the only constraint. Although precise figures are unavailable, it is known that PCI facilities differ in their capacity and not all of them operate 24-hour services. Moreover, the 60-minute target we have used takes no account of the reality that there may be substantial delays between onset of symptoms (occurrence of MI) and the patient starting the journey. These delays will reflect both knowledge and attitudes of patients and others around them, as well as the responsiveness of the emergency services if required. A study using data from the federal registry of acute coronary syndrome for the years 2009–11, covering 40 regions, reported that the median interval between the onset of symptoms and calling an ambulance was 158 minutes in 2009 and 134 minutes in 2011,^18^ whereas the median time taken for an ambulance to transport the patient to hospital was 55 minutes. In the recent registry of acute coronary syndromes RECORD-3 (covering the first 6 months of 2015, with 47 centres and 2370 patients), the median time from symptom onset until calling an ambulance was 3.4 hours—no better than in 2009–11.^19^

The regulatory framework in Russia requires that ambulance crews provide round-the-clock immediate medical assistance. The location and service (catchment) areas of the ambulance units are determined by population size and density, quality of road surface, intensity of traffic and taking into account the 20-minute transport accessibility. In 2015, in 87.0% of cases, the ambulance arrived within 20 minutes of the emergency call, but with substantial regional variability that could be caused by both real differences and report bias. However, these data are not available at a sufficiently disaggregated level to inform our analyses.

From a public-health perspective, there is the question of whether the sums needed to reduce travel time might be more effectively spent in other ways that would have a greater impact on reducing deaths from acute coronary syndrome. Yet, although this is important, there is also a question of equity. The principle of geographical equity of access to high-quality care is important and, although trade-offs may be necessary, it should be considered explicitly. Providing more PCI facilities could substantially reduce geographic inequalities across Russia.

Ideally, we would wish to see whether improvements in access times are related to declines in mortality. Certainly for Russia as a whole, there has been a reduction in the age- standardized death rate from MI for both sexes combined from 39.7 to 34.4 per 100 000 over 2010–15. However, there are many other potential contributors to this fall, including some evidence of a decline in smoking. The most informative analysis would therefore be to examine whether mortality declined more for the sections of the population who had improvements in nominal access times. Unfortunately, in Russia, the validated small-area mortality data that would be required are not available.

Some of these issues are being investigated in another part of the same project where we are looking at the experience of over 1100 AMI cases from 13 Russian regions. However, our current findings alone show clearly the scale of the challenges involved and have policy implications. First, despite the evident increase in the number of PCI facilities over the last decade, their current number and distribution appear to be insufficient, particularly in the southern part of European Russia, the Volga region and the Urals, where the population density is lower. Only by creating new PCI facilities, as reported in our subsidiary analysis, could Russia approach Australia and Canada in terms of the share of people living within 60 minutes’ travel access. Second, it seems reasonable to develop inter-regional collaboration, although this will require a new approach to funding healthcare. This would allow patients to go for treatment to the neighbouring region if that is where the closest hospital is located.

Finally, faced with limited resources, policymakers will have to set priorities for expenditure on new facilities. The approach that we have used, using data on the population that might benefit from expansion of services, can inform these decisions. In some cases, such as regions with small, sparsely populated settlements, it may be better to deploy advanced support in vehicles or non-PCI hospitals with subsequent transportation to PCI facilities by road or air if needed.

## Funding

This work was supported by a Wellcome Trust Strategic Award (100217/Z/12); the Norwegian Ministry of Health; the Norwegian Institute of Public Health; UiT, the Arctic University of Norway; and the Russian Academic Excellence Project ‘5–100’.

## Supplementary Material

Supplementary Data
