# Supplementary material for: Reducing geographic inequalities in access times for acute treatment of myocardial infarction in a large country: the example of Russia
Source: Int J Epidemiol. 2018 Aug 1;47(5):1594–602. doi: 10.1093/ije/dyy146 (PMC6208271; doi:10.1093/ije/dyy146)
Supplement: Supplementary Data [file dyy146_suppl_data.pdf]

SUPPLEMENTARY MATERIAL

Figure S-1. Modelling driving time to the closest PCI facility *within the region* (left) and to any closest PCI facility *ignoring administrative borders* (example of Bashkortostan, 2015).

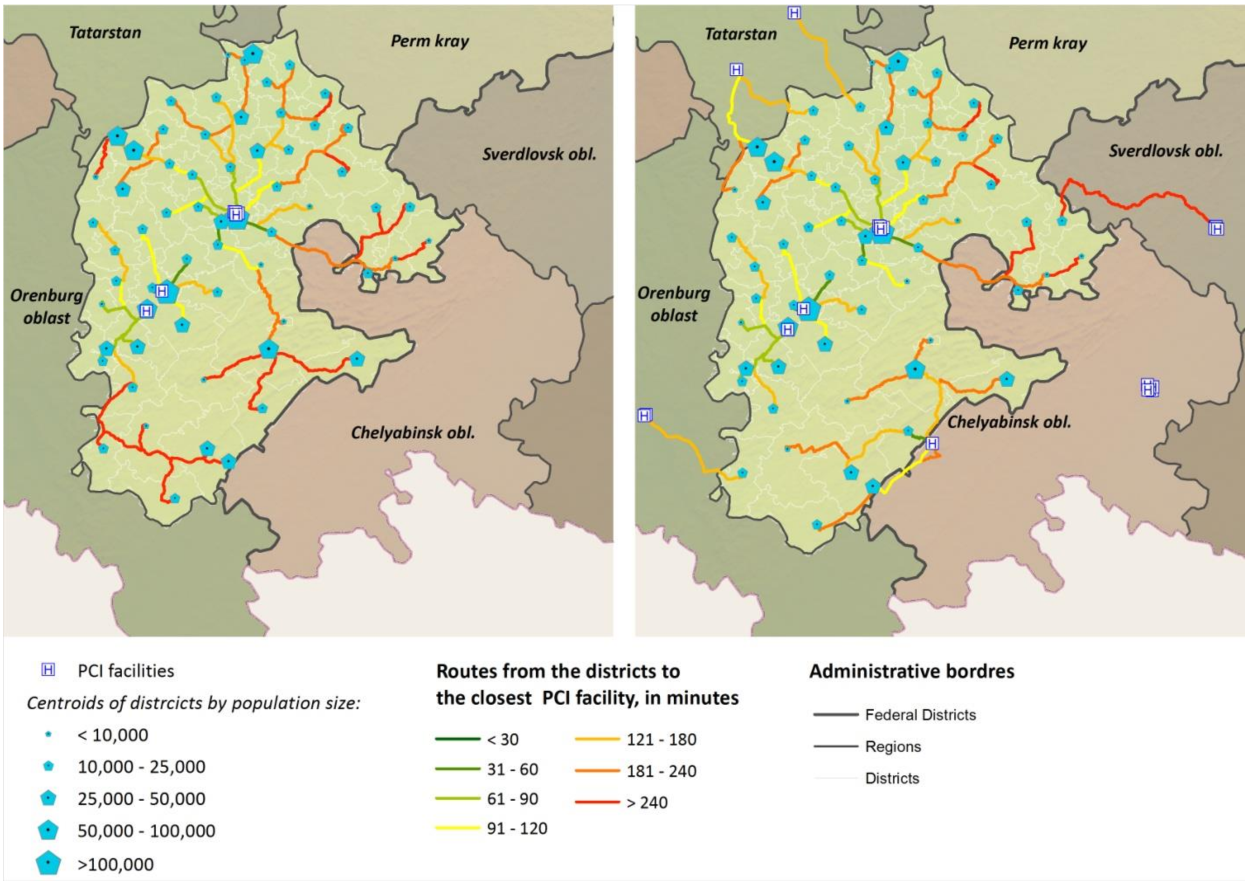

Figure S-2. Proportions of adults 40+ years lived within 60 minutes driving time to the closest PCI facility in 2015

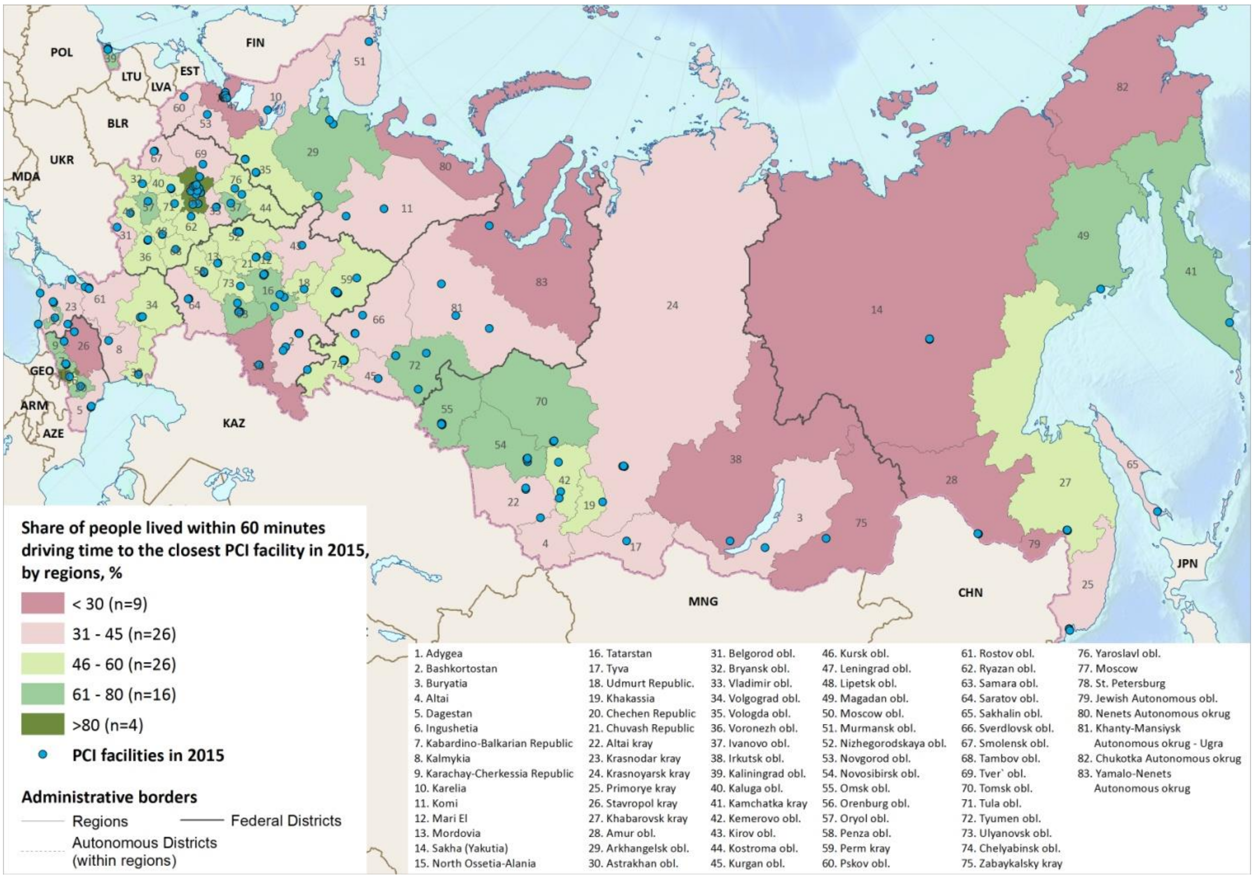

Figure S-3. Districts of Russia by the location of the closest PCI facility in 2015

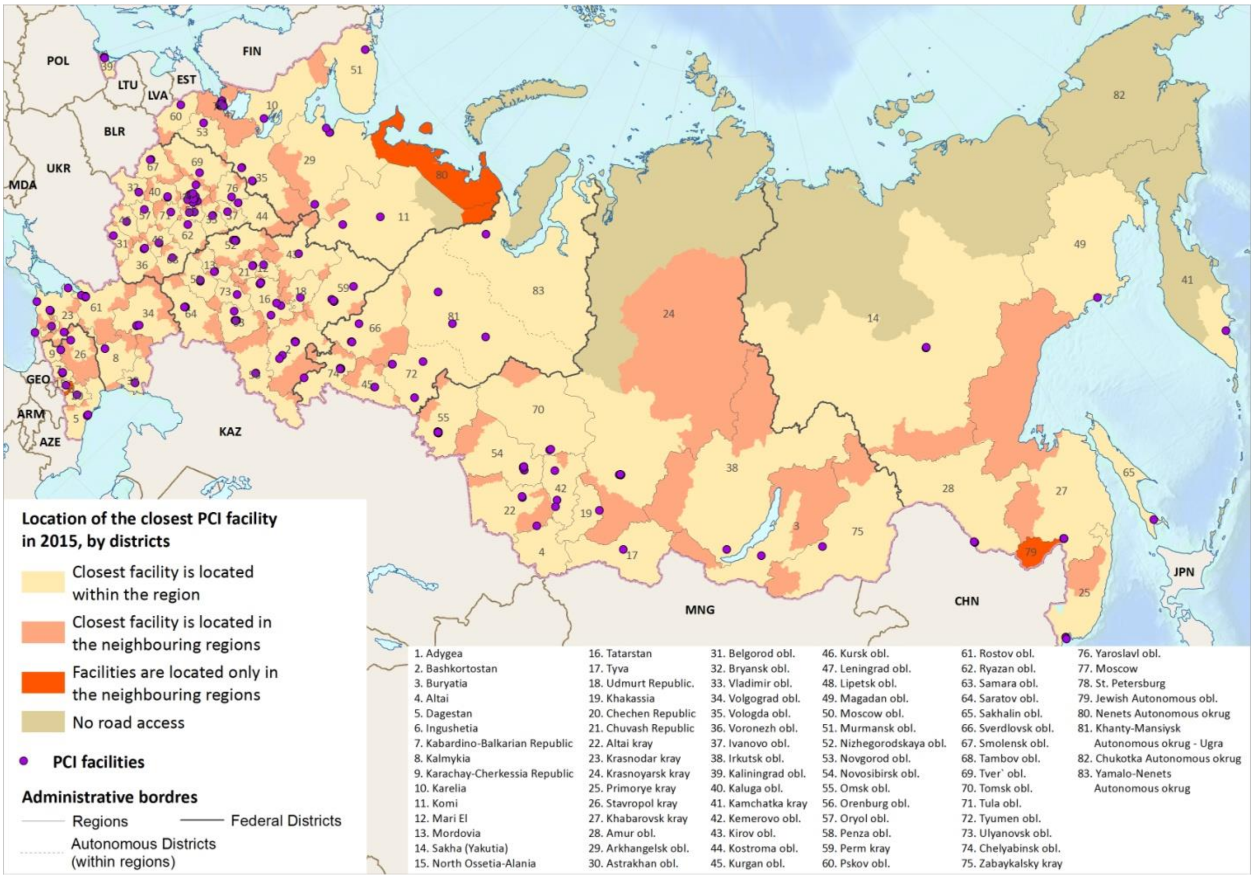

Figure S-4. Proportions of adults 40+ years who could live within 60 minutes driving time to the closest PCI facility

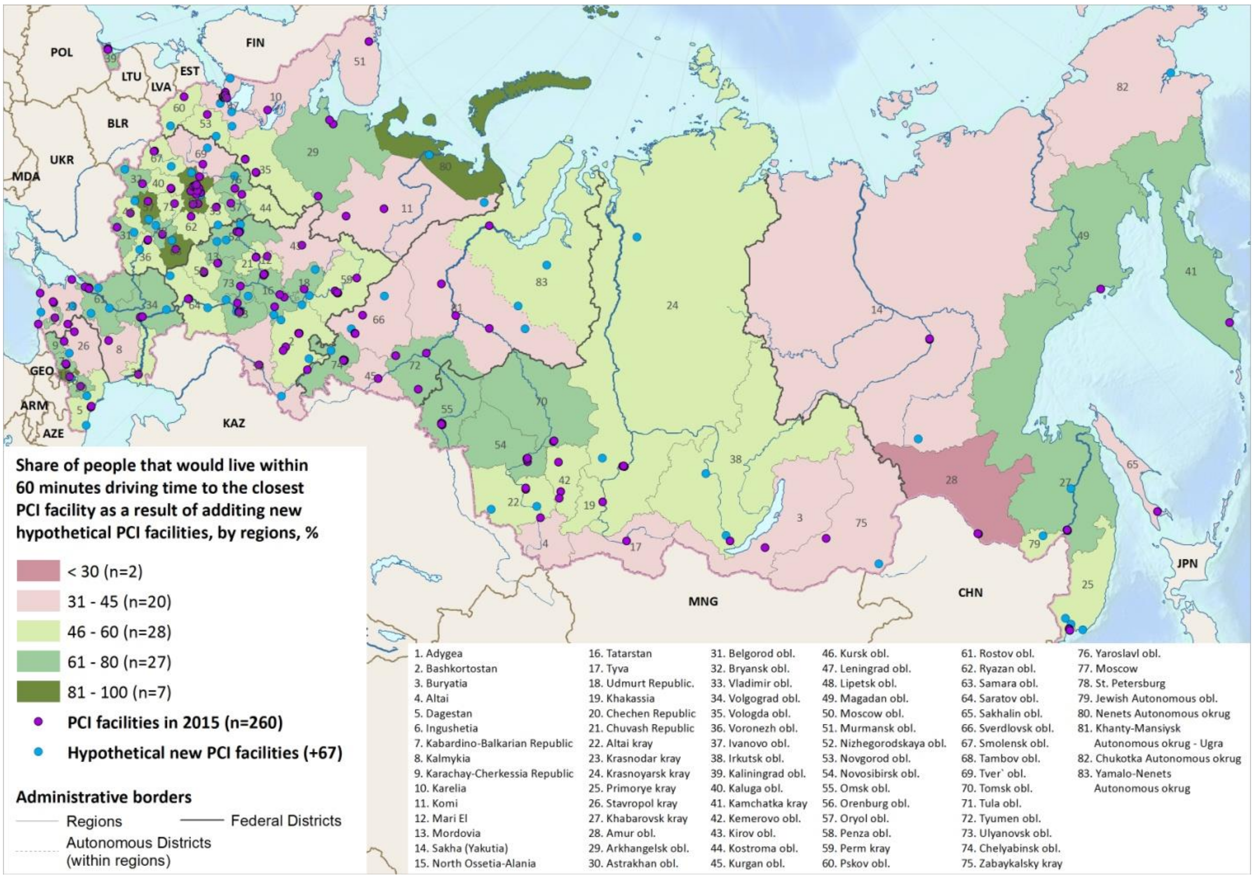

Table S-1. Selected indicators of timely access to the PCI facilities in federal districts, 2010 and 2015

Federal districts	Adults aged 40+ years, mln	2010				2015			
		Median time (IQR), mins	60 mins access, %	120 mins access, %	No road access, %	Median time (IQR), mins	60 mins access, %	120 mins access, %	No road access, %
**Central**	19.4	23.9 (9.5-83.3)	64.3	86.1	0.0	14.9 (7.2-74.2)	69.1	89.6	0.0
**North-West**	6.7	23.4 (7.2-120.1)	58.1	74.9	1.2	14.8 (6.8-104.8)	61.9	78.3	0.6
**Volga**	14.4	80.1 (14.1-141.2)	40.9	67.3	0.0	58.3 (11.5-124.8)	50.3	73.2	0.0
**South**	6.7	105.2 (19.9-173.8)	30.8	53.5	0.0	75.2 (8.6-126.7)	43.2	72.0	0.0
**North Caucasus**	3.5	108.7 (62.2-165.6)	22.7	56.1	0.0	79.7 (31.4-117.2)	41.3	76.7	0.0
**Ural**	5.5	100.2 (8.8-168.6)	37.8	60.5	0.6	79.4 (8.4-134.8)	43.5	69.0	0.0
**Siberian**	8.6	121.9 (19.3-282.8)	35.5	49.2	1.0	71.3 (11-221.8)	45.8	60.6	1.0
**Far East**	2.8	245.9 (37.6-657.9)	30.0	32.3	7.1	159.4 (34.6-404.8)	38.8	42.9	2.6

Table S-2. Improvements in timely access to advances medical treatment in federal districts by creating 67 new PCI facilities

Federal districts	Adults aged 40+ years, mln	The hypothetical conditions (creating 67 new PCI facilities)			Changes in comparison with 2015			
		Median time (IQR), mins	60 min access, %	120 min access, %	Number of added hospitals	Decrease in median time, mins	Increase in 60 mins access, percent points	Increase in 120 mins access, percent points
**Central**	19.4	13.4 (7.1-59.8)	75.1	94.6	13	1.5	6.0	5.0
**North-West**	6.7	13.2 (6.7-83.3)	68.0	83.3	8	1.6	6.1	5.0
**Volga**	14.4	35.1 (9.9-97)	60.3	84.5	16	23.2	10.0	11.3
**South**	6.7	55.1 (8.6-90.4)	52.7	84.3	6	20.2	9.5	12.2
**North Caucasus**	3.5	55.8 (13.9-84.9)	56.2	85.0	4	23.9	14.9	8.3
**Ural**	5.5	58.9 (9.0-116.6)	51.3	75.8	6	20.4	7.8	6.8
**Siberian**	8.6	47.3 (11.1-163.2)	53.5	68.3	7	24.1	7.7	7.7
**Far East**	2.8	52.6 (15.9-235.3)	51.6	63.1	7	106.7	12.8	20.2
